# New Instant Digital Pathology for EUS/EBUS Samples: The Last Advance in Bedside Diagnostics for Lung Carcinoma

**DOI:** 10.3390/cancers16234015

**Published:** 2024-11-29

**Authors:** Francesco Maria Di Matteo, Serena Stigliano, Luca Frasca, Dario Biasutto, Giulia Maricchiolo, Vittoria Morano, Chiara Taffon, Anna Crescenzi

**Affiliations:** 1 Operative Endoscopy Department, Fondazione Policlinico Universitario Campus Bio-Medico, 00128 Rome, Italy; f.dimatteo@policlinicocampus.it (F.M.D.M.); d.biasutto@policlinicocampus.it (D.B.); g.maricchiolo@policlinicocampus.it (G.M.); 2 Unit of Thoracic Surgery, Fondazione Policlinico Universitario Campus-Biomedico, 00128 Rome, Italy; l.frasca@policlinicocampus.it; 3 Unit of Endocrine Organs and Neuromuscular Pathology, Fondazione Policlinico Universitario Campus Bio-Medico, 00128 Rome, Italy; v.morano@policlinicocampus.it (V.M.); anna.crescenzi@uniroma1.it (A.C.); 4 Pathology Unit, Fondazione Policlinico Universitario Campus Bio-Medico, 00128 Rome, Italy; c.taffon@policlinicocampus.it; 5 Department of Radiological, Oncological and Pathological Sciences, Sapienza University of Rome, 00161 Rome, Italy

**Keywords:** lung carcinoma, endoscopic ultrasound (EUS)/fine needle aspiration/biopsy, endobronchial ultrasound (EBUS)/fine needle aspiration/biopsy, ex vivo fluorescence confocal microscope (FCM), instant digital pathology, adequacy assessment, real time diagnostics

## Abstract

**Background**: Ex vivo fluorescence laser scanning microscopes (FCMs) allow digital tissue imaging directly from fresh, unfixed specimens without the need for conventional histological slide preparation. To date, no data have been reported on the use of FCMs in the endoscopy suite for immediate evaluation of endoscopic ultrasound (EUS)/endobronchial ultrasound (EBUS) fine needle aspiration/biopsy (FNA-B) specimens of lung lesions and/or mediastinal lymph nodes. **Objectives**: The aim of this study was to evaluate the performance of the FCM Vivascope 2500 (Vivascope, Munich, Germany) in providing real-time adequacy assessment and diagnostic information on the digital images of fresh unprocessed EUS/EBUS FNA-B specimens and to compare it with the corresponding final histological sections of formalin-fixed and paraffin-embedded cell blocks. ** Methods and Results**: Thirty-two patients (50% male; 71 ± 8 years old) were enrolled between May 2023 and June 2024. In 28/32 (87.5%) patients, samples were defined as adequate at Vivascope evaluation, and in 20/28 (71.4%) patients, samples were classified as malignant. At final cytohistological evaluation, 87.5% of specimens were defined as adequate and 20/28 (71.4%) were diagnosed as malignant. There was perfect agreement between the Vivascope assessment of adequacy and the final cytohistological assessment on the same specimen (k Cohen 1). From a diagnostic point of view, perfect agreement was found between the two techniques in the identification of malignant neoplasms (k Cohen 1). **Conclusions**: The use of FCM could provide rapid information on both the adequacy and malignancy of the sample obtained during EBUS tissue acquisition (EBUS-TA), with minimal or no preparation and without destroying or losing the tissue. This technique allows for obtaining representative material in EBUS/EUS for lung cancer staging and is expected to change the turnaround time from biopsy sampling to diagnostic characterization of the tumor, ultimately improving patient management both at diagnosis and at restaging in follow up.

## Simple Summary

Advances in instrumentation technology have led to the development of new optical platforms for digital pathology imaging directly from fresh, unfixed specimens, without the need for conventional histological slide preparation. The objective of this study was to evaluate the performance of the new Vivascope 2500 digital microscope in providing real-time adequacy assessment and diagnostic information for echo-endoscopic lung and mediastinal nodes biopsy specimens. Thirty-two patients undergoing an EUS/EBUS procedure for the diagnosis of lung masses or lymph node staging were enrolled in this study: in 87.5% of patients, the specimens were adequate, and the immediate diagnostic hypothesis was a malignant lesion in 71.4%. When compared with paired permanent paraffin sections, there was excellent concordance between immediate digital assessment and final conventional diagnosis. Moreover, all digital real-time negative cases were confirmed as negative on permanent paraffin sections. The use of this approach provides rapid information on both the adequacy and malignancy of the sample obtained in real time during EBUS tissue acquisition, with minimal preparation and without destroying or losing the tissue. Real-time diagnosis with tissue preservation is expected to reduce turnaround time for diagnostic report and ensure adequacy for molecular profiling of the tumor.

## 1. Introduction

Real-time digital microscopy tools are emerging for rapid microscopic evaluation of cells and tissues, overcoming the limitations of conventional digital pathology. Recent advances in instrumentation technology have enabled the development of new optical platforms that allow digital tissue imaging directly from fresh, unfixed specimens, without the need for conventional histological slide preparation. Ex vivo fluorescence laser scanning microscopes (FCMs) use laser sources to achieve the acquisition of histological digital images through the interaction of photons with labelled and unlabeled tissue components. Proprietary software generates the resulting digital images in colors similar to hematoxylin and eosin (HE) staining in a very short time frame, typically less than one minute, without the need to freeze or section tissue [1]. The strengths of this technology include rapid digital imaging and complete preservation of the specimen for all subsequent diagnostic and ancillary techniques, which is generating great interest in various fields of pathological diagnostics. FCM is rapidly emerging as a powerful tool for intraoperative assessment of surgical margins, lymph node status and tumor mass, for rapid evaluation of core biopsies from prostate, breast, kidney and liver lesions [2,3,4] and more recently for cytological specimens and microhistological fragments from solid and cystic lesions of the pancreas [5,6]. Lung cancer is the leading cause of cancer-related mortality worldwide, accounting for approximately 1.2 million deaths each year: non-small cell lung cancer (NSCLC) accounts for the majority of cases [7]. Spread to ipsilateral (N2) or contralateral (N3) mediastinal lymph nodes precludes surgery as a first-line treatment, so accurate staging is required to properly plan patient management. Endobronchial ultrasound-guided transbronchial needle aspiration (EBUS-TBNA) and endoscopic ultrasound-guided fine needle aspiration (EUS-FNA) are minimally invasive alternatives to mediastinoscopy for the diagnosis and staging of mediastinal pathology [8]. This approach is suggested for lung tumor staging both in patients with radiological evidence of mediastinal nodal involvement and in those at increased risk due to hilar or central lung tumors [9]. The EBUS-TBNA procedure shows high sensitivity and specificity. Several systematic reviews and meta-analyses have reported the yield of nodal staging for non-small cell lung cancer by EBUS-TBNA to have a pooled sensitivity of 88–93% and a pooled specificity of 100% [10].

For most patients with stage N2 or above, surgery is often not indicated, and biopsy material is the only source of information available for tumor identification and subtyping, molecular profiling and immune checkpoint assessment [11]. Therefore, it is essential that sufficient material is available for all diagnostic needs at the time of EUS/EBUS. Rapid on-site evaluation (ROSE) of material obtained during the EUS/EBUS procedure has been proposed to assess the adequacy of diagnostic material, but ROSE is time consuming and resource intensive and is not performed or available in many centers [12]. To date, no data have been reported on the use of instant digital pathology microscopes in the endoscopy room for immediate evaluation of EUS/EBUS FNA-B specimens from lung lesions and/or mediastinal nodes. The aim of this study was to evaluate the performance of the FCM Vivascope 2500 (Vivascope, Munich, Germany) in providing real-time adequacy assessment and diagnostic information on the digital images of fresh unprocessed specimens and to compare the instant reports with the corresponding final histological sections of formalin-fixed and paraffin-embedded cell blocks.

## 2. Materials/Subjects and Methods

**Figure 1:**
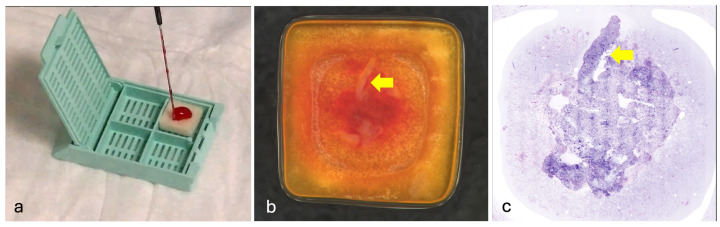
Vivascope protocol for obtaining digital images of EUS/EBUSA/FNAB samples of lung tumors or mediastinal nodes. (**a**) The fresh unfixed specimen was directly expelled onto a polymeric scaffold immediately after sampling. (**b**) The macro image shows the loaded scaffold: it is possible to identify small core of tissue (arrow) included in the sample. (**c**) Same sample. After digital acquisition, a whole digital slide of the specimens is available for the morphological examination. The core of tissue is visible in the image (arrow).

This study is part of the PNRR-POC-2022-12376531 project funded by the Italian Ministry of Health. The study protocol was approved by the Ethics Committee of the Fondazione Policlinico Universitario Campus Bio-Medico of Rome (Committee reference number N. SC 2022.205, date 21 December 2022, Fondazione Policlinico Campus Bio-Medico). In this prospective study, all enrolled patients were asked to sign the informed consent module. The study and sample processing were performed in accordance with the Declaration of Helsinki. All consecutive patients admitted to the Fondazione Policlinico Universitario Campus Bio-Medico of Rome for EUS or EBUS for lung cancer staging, between May 2023 and June 2024, were invited to participate in this study. Exclusion criteria were age under 18 years, mental disorders, pregnant women, co-morbidities hindering the EUS-EBUS procedure and refusal to sign the consent form. An Endoscopic Ultrasonography processor SU-1H FujiFilm (Japan) was used for all the procedures. The EB-530US Fujifilm scope (Fujifilm, Japan) was used for EBUS procedures and the EchoTip Ultra Cook (Cook Medical, Bloomington, IN 47402-0489, USA) needle was used for FNA. EUS was performed with a Fujifilm EG740UT linear EUS scope (Fujifilm, Japan) and an AcquireTM Boston Scientific needle (Boston Scientific Corporation, Marlborough, MA 01752-1234, USA) was used for FNB. The choice of the needle gauge was operator dependent usually linked with the lesion size and location. After the EUS/EBUS FNB procedure, the needle was removed from the endoscope and the sample obtained was ejected directly onto a polymer scaffold (Cytomatrix; UCS Diagnostic srl, Morlupo, Italy) (Figure 1a). The Cytomatrix serves as a holder to hold the specimen under the FCM objective, is paraffin compatible and retains both cellular material and tissue fragments during subsequent FFPE cell block preparation. Real-time digital imaging was performed using the FCM VivaScope^®^ 2500 (2500M-G4; VivaScope GmbH, Munich, Germany). Sample preparation was performed using a few drops of acridine orange to allow for digital visualization of cellular and subcellular microscopic details. The preparation protocol was carried out as previously described [13] and it took less than 10 min from sampling to obtaining the complete digital image of the specimen. The first image produced by the FCM Vivascope is the macro appearance of the loaded Cytomatrix, which allows for a preliminary assessment of the collected material (i.e., nuclear fragments, blood, fibrin clots, etc.) (Figure 1b). After this evaluation, it is possible to acquire the digital images by moving to the 40× objective with the laser sources. The reflectance modality highlights stromal components while fluorescence shows nuclear and cellular details. A proprietary algorithm converts reflectance to eosin-like red and fluorescence to blue-violet, producing full-image digital virtual slides with HE-like staining (Figure 1c). By moving along the *Z*-axis, it is possible to select the focal plane that best suits the diagnostic needs. For each enrolled case, one or more full-image digital slides were acquired and stored on the server. The pathologist evaluated the specimen by moving through the image and using a zoom of up to 500×. Immediate assessment of the adequacy of the specimen for both diagnosis and further analysis (i.e., at least 200 well-preserved neoplastic cells) was recorded in the database. A diagnostic opinion was also recorded, including differentiation between malignant and non-malignant processes, and a specific histotype if only morphologically recognizable. After Vivascope imaging, specimens were removed from the microscope slot and formalin fixed for paraffin embedding to obtain the final cell block. Permanent sections were stained with hematoxylin–eosin and immunohistochemistry was performed in cases requiring specific characterization (endocrine tumors, poorly differentiated adenocarcinomas). Pathologists were blind for the FCM results at the time of the paired conventional histological examination. The adequacy of the specimens and the diagnoses reported in real time on digital imaging were then compared with those obtained by light microscopy on permanent sections. Microscopic morphology on digital FCM images and on permanent sections was also observed by pathologists to assess the reliability of instant digital pathology in identifying a number of cellular and subcellular details for lung cancer diagnosis; these diagnostic clues included nuclear atypia, nucleolus evidence, cytoplasmic features, necrosis and mitotic activity. Statistical analyses were performed using IBM SPSS software v20 version (IBM Corporation, New York, USA). Quantitative data are expressed as medians and ranges, whereas categorical variables are expressed as numbers and percentages or frequencies. *p* < 0.05 was considered statistically significant. The weighted kappa statistic test (quadratic weights) was used to quantify the agreement between the FCM assessment and the final histological diagnosis on the same specimen [14]. Accuracy, sensitivity, specificity, positive predictive value and negative predictive value were calculated with 95% confidence intervals (CIs) using the histopathological diagnosis as reference.

## 3. Results

Thirty-two patients (50% male; 71 ± 8 years old) were enrolled between May 2023 and March 2024. Most patients (29/32, 90.6%) underwent EBUS FNA, 4/29 (13.8%) for lung mass and 25/29 (86.2%) for lymph node staging; the remaining patients (3/32, 9.4%) underwent EUS FNB (3/3, 100%) for lymph node staging. A 22 G needle was used in 27/32 patients (84.3%). In 28/32 (87.5%) patients, the specimen was defined as adequate on Vivascope evaluation and the diagnosis was reported as malignant in 20/28 (71.4%) patients. At final FFPE histological evaluation, 87.5% of the specimens were defined as adequate and 20/28 (71.4%) were diagnosed as malignant. The complete data set of the cases included in this study and the results of the morphological assessment are summarized in Table 1. The histological characterization is shown in Table 2. As evidenced in the table, there was perfect agreement between the Vivascope assessment of adequacy and the final cytohistological assessment on the same specimen (k Cohen 1). From a diagnostic point of view, there was perfect agreement between the two techniques in identifying cases of malignant neoplasm (k Cohen 1). The overall sensitivity, specificity, positive predictive value, negative predictive value and accuracy of the Vivascope evaluation were (100% 95% CI 83.2–100; 100% 95% CI 73.5–100; 100% 95% CI 83.2–100; 100% 95% CI 73.5–100%; 100% 95% CI 89.1–100). Diagnostic subtyping at Vivascope evaluation was achieved in 17 of 20 malignant lesions, while three cases were reported as NSCLC-NOS. The latter were diagnosed as poorly differentiated adenocarcinoma in two cases on permanent cell block sections with the support of immunohistochemistry, while one case remained NSCLC-NOS. Morphologically, nuclear atypia, nucleolus evidence and the presence of necrotic debris were easily identified in the Vivascope images. Cytoplasmic features in FCM images appear usually clear in adenocarcinomas and showed dense texture and pearl of keratin in squamous cell carcinoma, supporting the diagnosis of specific histotype. Mitotic activity was less evident in the Vivascope images than in the formalin-fixed sections.

## 4. Discussion

**Figure 2:**
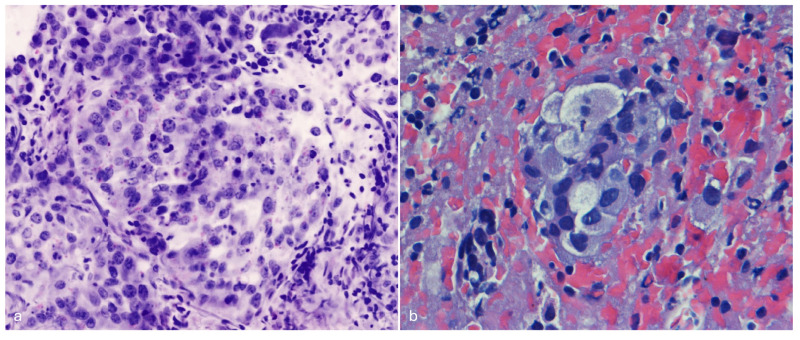
Metastasis of lung adenocarcinoma in a mediastinal node. (**a**) FCM image show malignant cells with irregular nuclei, evident nucleoli and faint stained cytoplasm, arranged in solid group with pseudo-acinar features. Intense neutrophilic inflammatory background is evident. (**b**) Permanent paraffin section from the same case. Malignant cells are arranged in cluster with pseudo-acinar growth. The bloody and inflammatory background is more evident.

**Figure 3:**
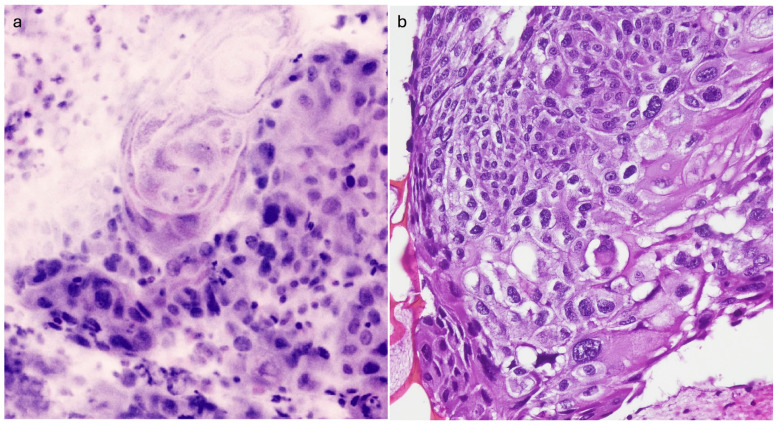
Metastasis of squamous cells carcinoma in a mediastinal node. (**a**) FCM image shows clusters of malignant cells with hyperchromic irregular nuclei of variable size and dense cytoplasm. A focus or keratinization (keratin pearl) is evident in the middle of the field. (**b**) Permanent paraffin section from the same case. Malignant squamous cells of variable size show dark nuclei and dense cytoplasm with evident keratinization.

**Figure 4:**
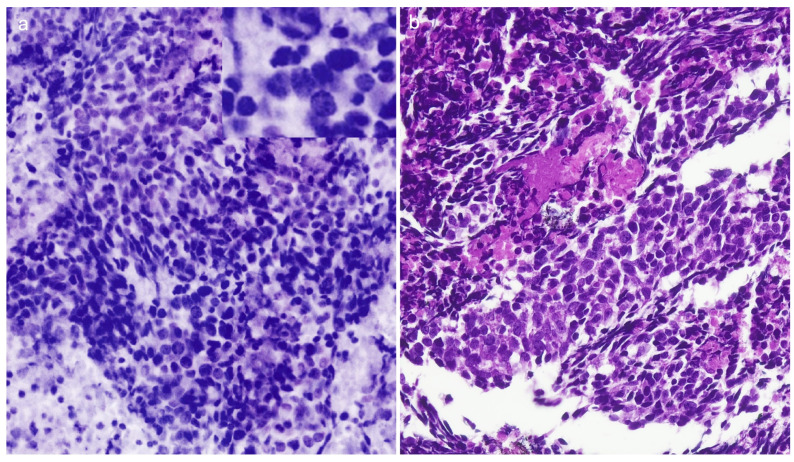
Small cell carcinoma of the lung hilum. (**a**) In FCM image, malignant cells of small size are arranged in loose sheets and show round nuclei with fine chromatin, no evidence of nucleoli, and scant cytoplasm. Nuclear molding is recognizable (inset). (**b**) Permanent paraffin section from the same case. Malignant small cells are loosely cohesive and associated with nuclear crashing and necrosis. Immunohistochemistry for neuroendocrine markers resulted positive.

**Figure 5:**
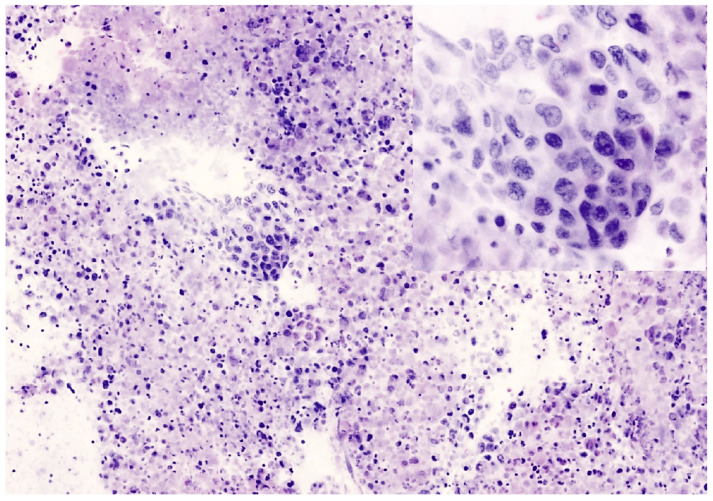
Metastasis of lung adenocarcinoma in mediastinal node. In this case there was a heavy necrotic and bloody background. The patient had a previous sampling reported as inadequate. FCM image allows fast identification of neoplastic cell clusters (inset) due to the unstained blood cells and cytoplasm debris resulting from the Vivascope protocol. This feature is specific of Vivascope imaging.

FCMs with optical imaging modalities have a promising potential for the evaluation of unprocessed cells and tissues in surgical pathology practice and can be considered as the next generation of digital pathology. FCMs truly represent an extraordinary technological advancement in pathology diagnostics, and promising results in surgical pathology of lung cancer have recently been reported [15]. In the present study, we describe for the first time the application of the FCM Vivascope in the evaluation of EUS/EBUS specimens in the diagnosis of advanced lung cancer and report a perfect agreement between the Vivascope evaluation of specimen adequacy and the final FFPE evaluation of the same specimen. Furthermore, when looking at the diagnostic field, there was a high concordance in the identification of malignant tumors between the two techniques. The first relevant advantage of optical sectioning microscopes is that the images are entirely digitally native. Avoiding any of the steps normally required to prepare conventional slides, FCMs generate digital images directly from the tissue or cells in a very short turnaround time, typically less than ten minutes from receipt of the sample to the complete image being made available [16]. Such images can be easily shared between pathologists, stored, retrieved and integrated into electronic health records. Notably, FCM digital images have less impact on server storage than conventional digital pathology images obtained from scanners, as a complete FCM digital image requires approximately 800 megabytes using a 40× objective, compared to the range of 5.0 megabytes—7.3 gigabytes for a scanned conventional slide using a 20× objective [17]. These characteristics make the FCM technology less expensive than digital pathology with digitized slides. Our data show excellent agreement between real-time Vivascope imaging and permanent FFPE sections in immediate reporting of specimen adequacy. Bedside adequacy information in the endoscopy suite allows the endoscopist to limit biopsy passes, thereby reducing the risk of procedure-related adverse events [18,19]. Adequacy assessment without loss or damage of diagnostic material is one of the most important aspects of the use of FCM in EUS/EBUS diagnostics. In fact, obtaining representative material from mediastinal and pulmonary lesions could be difficult due to the risk of obtaining samples with normal bronchial epithelium or blood contamination, resulting in a non-diagnostic report [20]. With the introduction of this new technology, pathologists will be able to visualize the neoplastic component, if present, in real time and ensure an adequate amount of tumor cells for molecular analysis, irrespective of the diagnosis. This is particularly relevant for mediastinal nodal restaging after neoadjuvant therapy, where the morphology of the tumor cells is more difficult to interpret and the demonstration of an adequate number of neoplastic cells for additional techniques is an important piece of information for the management of persistent disease [21]. In particular, the digital image obtained with FCM includes the entire amount of cellular material and tissue cores with the possibility of multi-level assessment and digital zoom up to 500×. Using FCMs, the accessibility of multi-level evaluation moving along the *Z*-axis corresponds to a serial sectioning of the specimen and is available for a minimum thickness of 200 microns, thus increasing the observable area of the specimen. This inherently 3D digital tool represents a significant advantage over both conventional histological sections and digital pathology slides. The FCM approach guarantees the evaluation of the almost complete volume of the EUS/EBS FNAB specimen, so that a broad agreement with FFPE sections can often be expected (Figure 2, Figure 3 and Figure 4). Samples from lymph nodes were reported as benign/negative for malignancy if a lymphocytic background, including histiocytes and plasma cells, without evidence of epithelial cells, was evident. All real-time negative cases were confirmed as negative on permanent FFPE. Thus, in the setting of negative specimens, the availability of whole specimen imaging with multi-level examination is key to achieving a correct diagnosis. Of note, the preparation of the sample to be observed at FCM is very simple and does not require specific training for technical personnel. Conversely, as a new tool for pathology practice, FCMs need to gain confidence with a new digital visualization modality, although the digital images are quite similar to those of digital pathology. Indeed, a previous study demonstrated a short learning curve for pathologists in the interpretation of FCM digital images in solid pancreatic masses [22]. Unlike conventional histological slides, erythrocytes are not stained with acridine orange, so they are not visualized in the Vivascope images and do not interfere with the identification of the cell population (Figure 5). We observed perfect concordance in the identification of malignant tumors between immediate assessment and final diagnosis. In the present study, we also report high concordance in the differentiation of small cell from non-small cell lung cancer and good agreement in the determination of tumor histotype. In fact, FCM images simulate the HE staining of conventional histology, and the microscopic images are easily comparable with morphological details that are diagnostic clues in lung pathology. In our study, nuclear atypia, evidence of nucleolus and presence of necrotic debris provided reproducible and robust morphological details. Of note, the nucleolus was usually evident, as expected for the nucleolus enhancement produced by acridine orange staining, due to the nucleolar avidity for acridine orange dye [23]. Cytoplasmic aspects were adequately identifiable and allowed us to recognize squamous cell carcinoma in this series (Figure 3). Indeed, nuclear features are reliable keys to identify malignancy and their association with cytoplasmic aspects supports the subtyping of lung tumors [24]. In this context, a limitation of FCM assessment is the possible difficulty in identifying the histological type in cases with poorly differentiated features. In fact, the FCM assessment is based on morphology only, whereas the exact classification of the cancer as adenocarcinoma or squamous cell carcinoma may require immunohistochemical characterization. As this classification is relevant in current practice of lung cytopathology diagnostics [25], specimens diagnosed as NSCLC-NOS on FCM images are sent to permanent cell block for immunophenotyping. In the present study, two out of three NSCLC-NOS diagnoses on Vivascope were finally reported as poorly differentiated adenocarcinoma after immunohistochemical assessment. Nevertheless, the adequacy of the sample for characterization is ensured by the immediate evaluation. Other limits of this study are the single-center source of the cases and a relatively small number of patients; future studies with larger numbers of cases are expected to confirm these results. As a digital pathology technique, FCM images can be shared online or remotely between pathologists, ensuring a high level of diagnostic service, even in hospitals without a pathology laboratory, and contributing to the development of skills in hospitals without specific diagnostic expertise. In recent years, growing evidence of new application of ex vivo confocal laser scanning microscopes is observed in various field of pathology diagnostics, supporting the wide interest for this technology [26,27,28,29]. Finally, artificial intelligence algorithms have recently been proposed for automated analysis to support diagnostic interpretation of FCM images [30].

## 5. Conclusions

Our study demonstrated a reliable performance of FCM in enabling diagnostics in the endoscopy room. This new generation of digital imaging appears to be suitable for routine use in lung pathology and is expected to meet the demands of modern diagnostics for speed, accuracy and completeness of data, especially in small samples of advanced tumors.

## Figures and Tables

**Table 1:** The complete dataset of the cases enrolled in this study and the results of the morphological assessment.

General Features	N (%)
**Male**	16/32 (50%)
**Mean Age**	71 ± 8
**Technique**	
*EBUS*	29/32 (90.6%)
*EUS*	3/32 (9.4%)
**Needle size**	
22 G	27/32 (84.3%)
25 G	5/32 (15.6%)
**Sample Site**	
Lymph node	28/32 (87.5%)
Lung	4/32 (12.5%)
**Vivascope evaluation**	
Adequate	28/32 (87.5%)
Diagnostic	28/32 (87.5%)
**Final cytohistological evaluation**	
Adequate	28/32 (87.5%)
Diagnostic	28/32 (87.5%)

**Table 2:** The diagnostic interpretation immediately after sampling using FCM Vivascope and the final diagnosis on the permanent paraffin sections.

Diagnosis	FCM Vivascope	Permanent FFPE
Negative for malignancy	7	7
Malignant	20	20
NSCLC (total)	17	17
Adenocarcinoma	13	15
Squamous cells carcinoma	1	1
NSCLC-NOS	3	1
SCLC	3	3

## Data Availability

The data are not publicly available due to privacy restrictions. The data presented in this study are available upon reasonable request from the corresponding author.
